# Embrace or leave social media? On the viability of public service media organizations’ strategies facing platform power

**DOI:** 10.1177/02673231241290097

**Published:** 2024-10-25

**Authors:** Hallvard Moe

**Affiliations:** 1658 University of Bergen, Norway

**Keywords:** public service broadcasting, platforms, Norway, news use, social media

## Abstract

This article addresses the question of whether a withdrawal from social media platforms represents a viable strategy in the ongoing relational power negotiations between public service media and platforms, and if so, under what conditions. Recent diverging strategic efforts from public service broadcasters in two European countries – Germany and Norway – serve as illustrations of how existing organizations attempt to manoeuvre the current online realm dominated by platforms. I discuss findings from qualitative studies of news use in Norway and draw on comparative survey data on news use. I propose that high levels of trust in public service media, and widespread use of the public service broadcaster's own sites and offers, are key prerequisites to consider when assessing the viability of a withdrawal from third-party platforms in different countries. By zooming in on one type of media organization (public service broadcasters), and looking at different such organizations’ characteristics and context, and the dynamics of their relations to online platforms, the paper contributes to our understanding of platform power.

## Introduction

Three decades have passed since the first European public service broadcasting organizations ventured on to the Internet. As organizations in different countries have built very different online presences under very different conditions, a point of debate has remained prevalent: The scope and form of public service media online (e.g. Bardoel & D’Haenens, 2008; Brevini, 2010; Donders et al., 2019; Enli, 2008; Holtz-Bacha, 2021). Recently, a key question has been whether broadcasting organizations should utilize the so-called platforms to reach new audiences or focus on their own site to build a clear public alternative.

Platforms are ‘data infrastructures that facilitate, aggregate, monetize, and govern interactions between end-users and content and service providers’ (Poell et al., 2022: 5). As critical scholars have shown, the platform metaphor glosses over as much as it reveals (Gillespie, 2010): Rather than a neutral basis for cultural and political communication, social media and other digital services that are termed platforms represent an enclosed, data-driven and ad-funded mainstream – features that run directly counter to the basic public good character of public service media. Scholars have argued that the degree of in-/dependence news organizations have vis-à-vis such platforms varies, creating different ‘spaces of negotiation’ for the relational power between these entities (Poell et al., 2023). The character of the media organizations matter here, and public service organizations have been held as well-positioned, being trusted news providers that do not have to worry about dwindling subscription and ad revenues (Poell et al., 2023: 14).

One might see relations between public service broadcasters and platforms as just another round in the ever-present debate about the role of these organizations, with shifting priorities leading to different positioning against new technologies and third-party actors in national policy struggles. But the question can also be an inroad to consider the context for public service media in Europe and how that context matter for organizations’ strategic leeway. Rather than intervening in such a policy debate to argue for a specific strategy, this article addresses the question of whether a withdrawal from social media platforms represents a viable strategy in the ongoing relational power negotiations between public service media and platforms and if so, under what conditions.

For this purpose, I first revisit earlier attempts at envisioning public service media online as an alternative to the mainstream. In contrast, recent diverging strategic efforts from public service broadcasters in two European countries – Germany and Norway – serve as illustrations of how existing organizations attempt to manoeuvre the current online realm dominated by platforms. While the German public service organizations’ initiative *funk* employs different third-party platforms to reach young audiences, the Norwegian example exemplifies an attempt at withdrawing from said platforms. Focusing on the viability of the latter move, I discuss findings from qualitative studies of news use in Norway and draw on comparative survey data from the *Reuters Institute Digital News Report 2023*. I propose that (1) high levels of trust in public service media and (2) widespread use of the public service broadcaster's own sites and offers are key prerequisites to consider when assessing the viability of a withdrawal from third-party platforms in different countries.

By zooming in on one type of media organization (public service broadcasters), and looking at different such organizations’ characteristics and context, and the dynamics of their relations to online platforms, the paper contributes to our understanding of platform power.

## Public service media online: From grand visions to concrete strategic dilemmas facing platforms

Visions for a new future of public service media tend to surface in tandem with the emergence of new media technologies. So also for the online realm. It is useful to note some of those visions, since they remind us of how scholarly debate has dealt with the possible futures of public service media in the digital era (Moe, 2008b).

Twenty years ago, taking what he saw as a disintegrating public as the starting point, Stephen Coleman believed the Internet could be a medium ‘for new relations between citizens’ (Coleman, 2004: 91) and an ‘authentic connection between people and their representatives’ (Coleman, 2004: 91). Coleman set out to create new arenas for ‘civilised public debate’. The main part of the solution was a ‘publicly-funded, independently-managed online civic commons’, in which he envisioned a ‘key role’ for public service broadcasters like the BBC (Coleman, 2004: 96, 98). Around the same time, Graham Murdock (Murdock, 2005) proposed a tangential model. Murdock also wrote about a British context and resituated the remit within what he called a ‘digital commons’: ‘a linked space defined by its shared refusal of commercial enclosure and its commitment to free and universal access, reciprocity, and collaborative activity’ (Murdock, 2005: 227). Murdock saw this space as potentially global in scope, with public service broadcasting making up ‘the central node’ in the network (Murdock, 2005: 227). Importantly, back then, the television set was assumed as the terminal for the digital commons (Murdock, 2005: 228). These two visions are ambitious on behalf of the public good, and both hold public service media organizations as key to release a democratizing potential of digital media technologies.

Two decades later, it is safe to say that none of these visions came to life. Instead, we have witnessed a massive growth of platforms (Nieborg & Poell, 2018), with strong concentration of audiences on social media services. These platforms – search engines and content services – are owned by a handful of commercial companies and they spearhead ‘surveillance capitalism’, which monetizes data from tracking users online (Zuboff, 2020). Coleman's call for building tools to facilitate communication between citizens and their representatives has been more or less wholesale taken up by different social media platforms. And a commons seems to be the antidote to existing structural conditions online. This is not to say the idea of the digital commons disappeared, though. It surfaces from time to time, for instance, in a recent EU policy initiative to strengthen ‘Europe's digital sovereignty’, highlighting communal production, open source and co-creation (e.g. Keller, 2022). Relatedly, more recent calls have been made for a ‘public service internet’ (Fuchs & Unterberger, 2021). But the all-dominant tune is played by surveillance capitalism, which in presupposing enclosure and built on the marketization of personal data, is the opposite of a digital commons and the idea of media as public goods.

The situation for media content providers is made more difficult due to constantly changing conditions offered by the platforms at the head of surveillance capitalism. Not only do new platforms emerge, rise in popularity and then fade away – MySpace is a well-known example, but there are plenty of smaller also nationally based cases. Specific platforms also frequently change their terms of use, governance structures and business priorities. The debacle surrounding Elon Musk's much-publicized takeover of Twitter/X is but one illustration. Facebook's changing algorithmic prioritization of, in chronological order, ‘social news’, ‘quality’ content and then posts from friends and family is another (Poell et al., 2023: 7). Under these conditions, European public service broadcasting organizations have attempted to manoeuvre the landscape of third-party platforms somehow, between the extremes of abstention and cooperation. To illustrate diverging recent strategic moves, I now turn to consider organizations from two European countries.

## Illustrating diverging strategic moves: Germany and Norway

Beyond scholarly radical visions, existing public service broadcasters had to deal with concrete regulatory and economic realities facing digital media in the 1990s and onwards. These realities were not unison across national contexts. German public service broadcasters were more severely limited in their exploration than comparable Western European sister organizations (Moe, 2011): Rather than acknowledging the innovative potential of the Internet for new forms of content and new modes of communication far beyond radio and television broadcasting, the regulatory regime for public service media organizations restricted use of the Web to merely extend their analogue offers. This logic was followed consistently to its fascinating extreme with the rule that content published online had to be ‘de-published’ after a set number of days (e.g. Gizinski, 2010). If any, this regulation symbolizes the clash of analogue and digital media logics.

Importantly, the national German press has been the key adversary of public service media – not global online platforms. Such platforms have been explored in various ways over time, but a recent initiative is interesting for the present discussion, since it can illustrate a clear-cut strategy of utilizing or embracing social media platforms.

*funk* is the name of the initiative launched by the two public service media organizations ARD and ZDF. Motivated by the difficulty of reaching young audiences, *funk* provides audio-visual content that is distributed and consumed on social media platforms – not on the public service organizations’ own websites. As of 2023, *funk* provided more than 70 ‘channels’ on different social media platforms. Stollfuß has documented the emergence of the ‘network’ and discussed it in relation to German media policy as well as platform power (Stollfuß, 2019, 2024). As he states, *funk* ‘must provide content that fits the social media ecosystem and it is forced to permit [social media platforms] to generate and influence an audience within their operational boundaries’ (Stollfuß, 2024: 189). Sollfuß reads this particular case as an illustration of a wider strategy where the ARD and ZDF appear ‘to be actively adapting to an institutional operational framework within a third-party platform-driven environment’, rather than ‘proposing alternative infrastructural conditions’ (Stollfuß, 2024: 199).

Around 2023, then, *funk* illustrates an ambitious attempt from well-established public service media organizations to cater to young audiences by way of platforms’ distribution power – embracing the possibilities that these commercial entities offer for content providers. While falling way short of illustrating a full-fledged such alternative, the recent strategic manoeuvre from the Norwegian public service media organization NRK can serve as a contrasting example.

When it comes to actual strategies, the NRK has not been consistent in their use of third-party platforms. As the media sector faced digitalization, the organization was granted freedom to experiment and test different new services, including digital television and web services (Moe, 2011). The history of the NRK since the late 1990s is littered with examples of small and bigger attempts at exploiting new platforms and genres as part of the remit, whether it is the early virtual world of *Second life* or basic computer games (Moe, 2008a), video blogging distributed through Facebook (Sundet, 2008), YouTube channels (Moe, 2008c) or work to facilitate personalization (Van den Bulck & Moe, 2018). As of 2023, the strategy swung towards a more careful use of such platforms.

At this point, the NRK communicated a line of public ‘withdrawals’ of offers from Facebook and Instagram. Accounts affiliated with different genres have been removed: NRK News, Sports and most recently, regional news services. In explaining the decisions, the NRK offered statements addressing the relations to the powerful platforms and the dilemma of negotiating with them: ‘this is a step in NRK's strategy to strengthen our own platforms and make us less dependent on third parties’ (in Weiberg-Aurdal, 2022), and ‘NRK needs strong platforms of its own – such as nrk.no, NRK Radio and NRK TV – that the public uses and trusts’ (MacGregor, 2022).

Importantly, these specific initiatives should not be interpreted as a clear-cut break from engagement with platforms. The NRK still provides a range of content on different third-party platforms, so the policy does not appear consistent. Even after banning employees from having TikTok installed on their phones, to pinpoint one controversial issue, the NRK hesitated (in 2023) to stop their content production for young people on TikTok (Aarli-Grøndalen, 2023). Still, there is a tone set with these withdrawals that resonates with more general discussions of how public service media can deal with platforms while still upholding their remit.

What these two brief examples of recent actual strategic moves show, though, is that there are obvious differences in how different media organizations of the same type – here, public service ones – manoeuvre in their negotiations of power with platforms. To further thinking of exactly what features that can matter for such differences, and to what extent and for which organizations a withdrawal might be a viable strategy, it is worth dwelling with the case of the NRK.

## The case of the NRK

In case study methodology, the NRK would count as an ‘extreme case’ in being unusual and ‘especially good in a more closely defined sense’ (Flyvbjerg, 2006: 230). The NRK developed in, and represents, a Nordic ‘media welfare state’ (Syvertsen et al., 2014). Today, the organization remains well-funded, collaborates fairly well with commercial national competitors and has seen no serious attacks from political actors seeking to severely limit its remit. This is important to keep in mind as we discuss its role.

Qualitative studies of news use can shed light on some aspects of how people consider the NRK news site, relevant to understand the upside of such a strategy. In a study of the news habits of young Norwegians who did not subscribe to digital news, we found that particularly the NRK's news site ‘was frequently mentioned as a prime source, valued as a free, high-quality, trustworthy news provision’ (Borchgrevink-Brækhus & Moe, 2023: 9). Informants described how they could turn to the NRK's website for information on issues they heard about elsewhere on- or offline, with the underlying notion that if the issue was important, the NRK would have decent coverage. The NRK site was a source used in a similar way as ‘municipality websites, and, during the Covid-19 pandemic, public health information sites’ (Borchgrevink-Brækhus & Moe, 2023: 11). This highlights the value of a public service media site as an open domain with quality content, as opposed to a spread-out dissemination machine.

In a follow-up study, Borchgrevink-Brækhus (forthcoming) probed news use further, also focusing on more tactile and habitual aspects. She found the NRK to be valued for ‘soberness’, not just in the journalistic style, but in layout and design. The NRK's front page was described by one informant, appreciating the black-on-white headlines without flashing colours, explaining: ‘It's because they present you with one article at the time [in the mobile version] instead of having many next to each other’ (in Borchgrevink-Brækhus forthcoming). What is more, other informants emphasized technical advantages like quick loading and no glitches or lagging. These aspects can be seen to shed further light on why and how a public service broadcaster's own site might be valued as a source for citizens.

On this basis, we might attempt to distil some features from the case of Norway that enable a broader discussion of the conditions for withdrawal from platforms across national contexts. A first feature that seem evidently relevant is the elusive notion of trust. A second feature that seems perhaps more directly relevant is use of news provision from public service broadcasters directly, as opposed to via third-party platforms. The relevance of these features also seems to resonate in the NRK's own presentation of their opportunities: As stated by a NRK social media advisor, ‘when more and more users seek out our own platforms directly, that means we can scale down our efforts in social media. We can rather spend our energy on offering the best possible content and a better user experience where the audience increasingly prefers it’ (MacGregor, 2022).

Clearly, trust and direct use of providers’ own website are not the sole features that decide the chances of succeeding with a withdrawal from social media's surveillance capitalism. The aim here is not to deliver simplistic strategic advice. Rather, by discussing difference across cases in terms of these two central features, we might get a better understanding of how context matter, and to which extent the extreme case of Norway has relevance for other public service media organizations. So, to discuss the viability of the strategy attempted by the NRK, I now proceed to compare findings on trust and news use from a survey of Europeans’ news use.

## A comparative perspective on the viability of challenging platforms

To assess the viability of the strategy presented around 2023 by the extreme case of NRK in Norway in contrast to the German case's embracement of platform distribution, we can consider findings on media trust and news use in different countries. More specifically, (1) levels of trust in public service broadcasters; (2) use of news directly from editorial news providers, as opposed to via third-party platforms. For this purpose, the *Reuters Institute Digital News Report 2023* (Newman et al., 2023) can provide data. The report builds on a yearly survey in collaboration with national partners across and beyond Europe, and the data was collected by YouGov with an online questionnaire in early 2023.^1^

Media trust is difficult to measure in surveys (Jakobsson & Stiernstedt, 2023; Kohring & Matthes, 2007): It is neither necessarily correlated to use nor clear what the object of trust is (news in general, specific journalists or brands for instance) for different respondents. These issues get amplified when comparing across cultures. Expressing in a survey question trust in authorities or public institutions such as the media might be very different depending on historically grounded cultural norms, but also recent more or less relevant societal events (also Knudsen et al., 2022). As recent studies show, news users employ shortcuts and tacit knowledge to make quick considerations of the trustworthiness of specific news stories (Ross Arguedas et al., 2024; Swart & Broersma, 2022). Especially for young users, an established and loyal relationship to specific brands is not always the most evident way to decide trust.

Reported trust levels in public service media organizations across Europe need to be interpreted with these conditions in mind. A general finding from the *Digital News Report* is that national public service media institutions tend to do well on rankings of trust levels relative to main competitors in their markets (Newman et al., 2023). Even if we do not compare specific levels directly, a take-away from these findings is that public service media institutions in media systems with a tradition for relatively stronger editorial independence, less interference from authorities and more stable financial conditions seem to be more trusted compared to competitors. So, for instance, in the Nordic countries and Northern Continental Europe, national public service brands do better in an internal national comparison than in Southern and Eastern parts of the continent (Newman et al., 2023).

**Figure 1.**
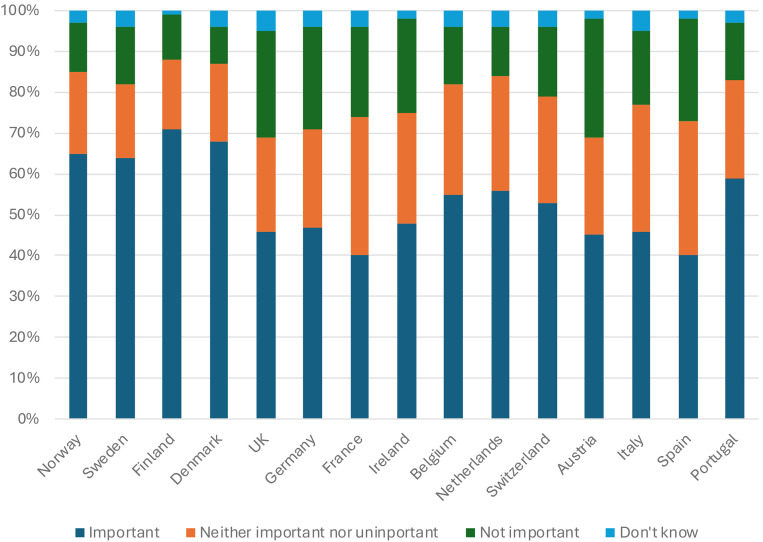
‘How important, or not, are publicly funded news services such as [country's public service media organization] to you personally?’. N = ca 2000 in each market. Digital News Report, 2023.

Another inroad to compare the standing, assumingly also the trustworthiness, of different public service media organizations is by way of how important specific organizations are for respondents personally. Figure 1 depicts a ranking based on such a question. A cluster of top-ranking countries (from the Nordic region) resonates with what we know about stable and favourable conditions, editorial independence and generous regulatory frameworks (e.g. Syvertsen et al., 2014), the remaining list also shows findings that are not easily explained by such features (e.g. Germany or the UK vis-à-vis Portugal).

While trust and personal preferences for public service media might not provide a very evident impression to assess the viability of platform strategies, then we can seek to clarify by way of findings concerning how people get to news.

**Figure 2.**
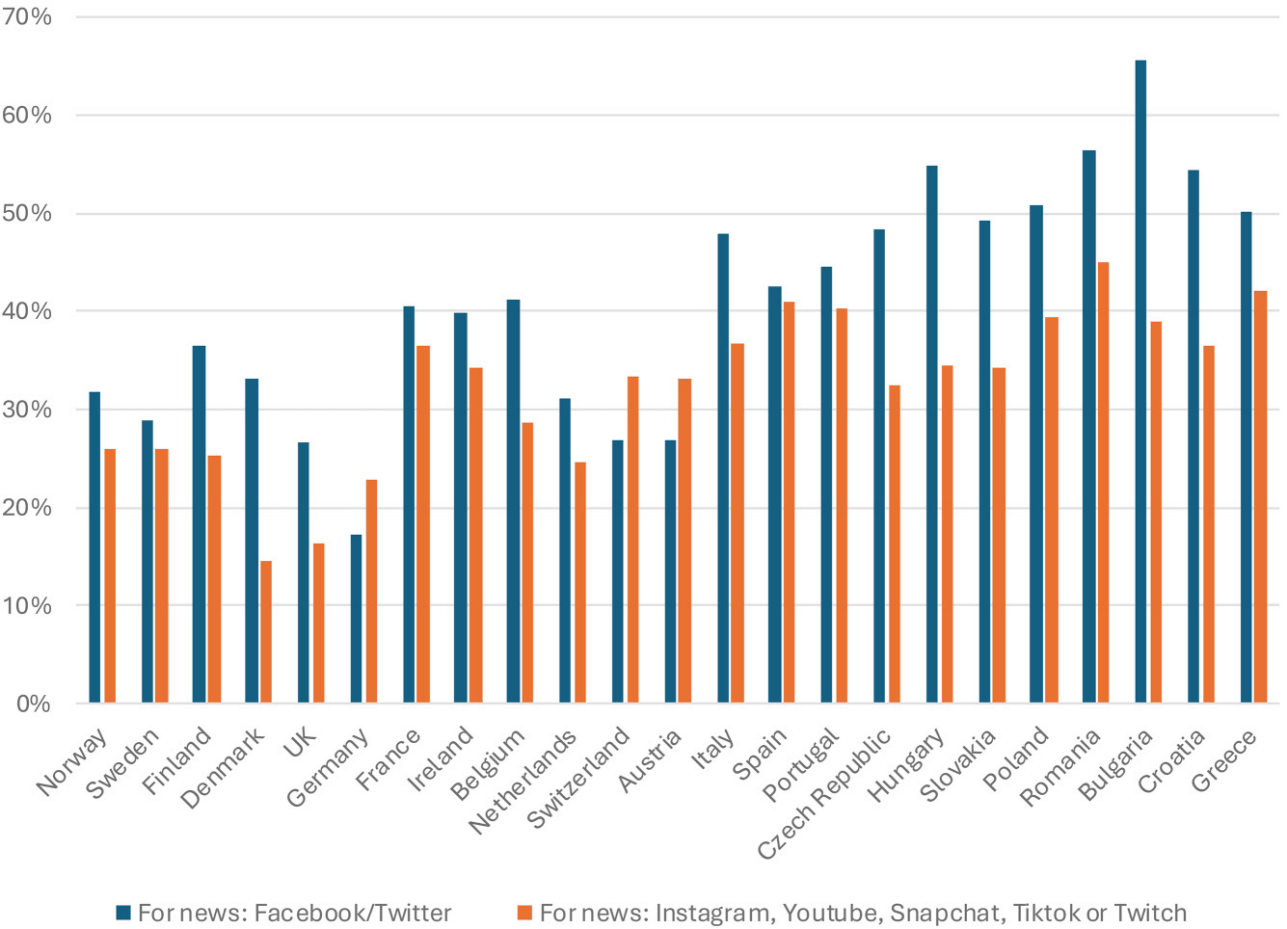
‘Which, if any, of the following have you used for finding, reading, watching, sharing or discussing news in the last week? Please select all that apply.’ Selected variables. N = ca 2000 in each market. Digital News Report, 2023.

 Figure 2 shows distribution across 23 European countries of habits of news use via different social media platforms. In the figure, countries from different parts of Europe are grouped together. There are deviations, but a general pattern that emerges is of relatively higher levels of use of social media platforms in Southern and Eastern European markets. For instance, 32% of Norwegians reported to have used Facebook or Twitter/X for news in the last week, and 26% said the same for video platforms including YouTube. The corresponding numbers are significantly higher in countries like Italy (48% and 37%) and Romania (56% and 45%). This is not to gloss over challenges in the better-faring countries, especially connected user groups with less financial leeway (e.g. in Norway, YouTube being more popular among households with lower income levels). Another challenge felt also in these well-faring cases is clearly younger audiences. In Norway, while those under 24 years of age consume *less* news than the average from Facebook and Twitter, they report significantly higher use of newer platforms for news: 37% for Snapchat and 24% for TikTok, compared to 11% and 6% for all respondents. In fact, more than half of all 18–24 years old in Norway claimed to have used one or more of Instagram, YouTube, TikTok, Snapchat and Twitch for news in a week. The takeaway is clearly that the dilemma of reaching young audiences – in our illustrations tackled so differently by the German and Norwegian organizations – is real also in the most extreme positive case.

What is more, on a general level, when close to half the population of online news users routinely access news through social media platforms, the outlook is rather bleak for any public service media institution that seeks to negotiate its independence from said platforms.

**Figure 3.**
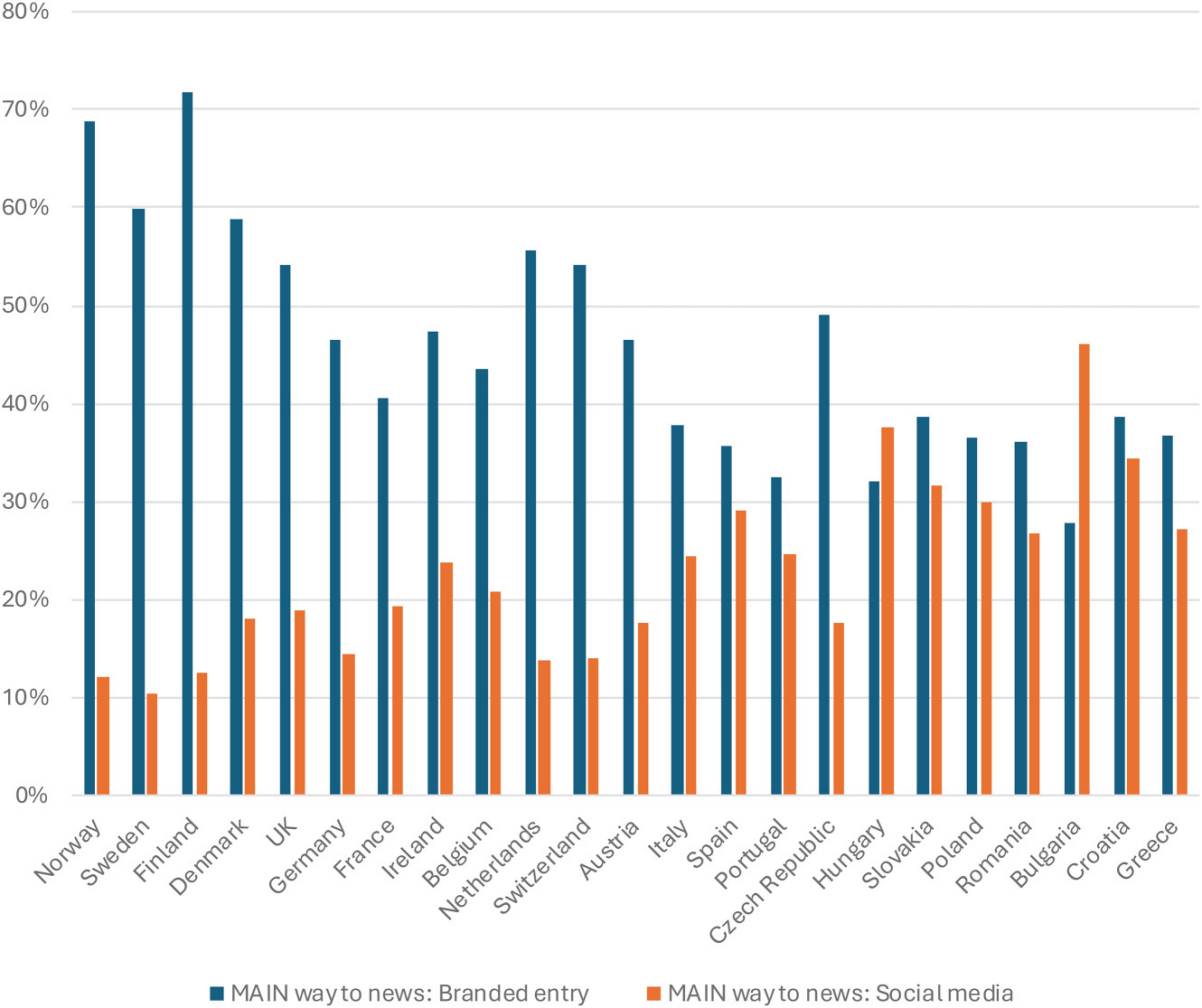
Filtered through ‘Thinking about how you got news online (via computer, mobile or any device) in the last week, which were the ways in which you came across news stories? Please select all that apply.’, ‘Which of these was the main way in which you came across news in the last week?’. *N* = ca 2000 in each market. Digital News Report, 2023.

An additional way to further scrutinize comparative differences on the issue of the relative importance of platforms for news is by asking about people's main gateway to news,

In Figure 3, the same 23 markets are surveyed. The branded entry-category consists of users going directly to a news provider's website or searching for specific news brands. The category ‘social media’ gathers larger and smaller, also nationally specific, platforms. The picture that emerges is again one with a clear division between the North and South/East of Europe. Editorial news represents the main way for routine news use for a clear majority in the former group of countries with, for example, 60–70% in the Nordic region, and above 40% in all Northern markets – compared to below 40% in the Southern and Eastern markets. Equally important, the preference for social media platforms as the main way to get to news is correspondingly higher in the markets to the South and East of the Continent (around 30% in several countries, 46% in Bulgaria) and lower in the Nordic region (10–18%). Based on this data, the relative position of any editorial news provider – public service media included – considering its policy vis-à-vis social media platforms, comes out as markedly different across different parts of Europe.

## Conclusion

The starting point for this article was the seemingly ever-present discussion of the role and remit of European public service media. In recent years, this discussion has been focused on the breadth of activities online, with the use of, or collaboration with, the so-called platforms as a focus point. In its current form, this debate encapsulates a principled dilemma: Through a range of different institutional forms, the idea behind public service broadcasting has always been to provide a public good for citizens. The enclosed, hyper-commercial and privately controlled environment created by the online platforms represent the antidote, while simultaneously offering a much-needed way to reach new and larger groups of citizens.

As work on power dynamics online has pointed out, it makes sense to approach the situation as creating a space of negotiation between media organizations and platforms and to view this space and the relational power balance as different based on forms of media organizations (Poell et al., 2023). The discussion of recent strategic choices made by public service media organizations in Germany and Norway served to illustrate diverging paths. The German *funk* network seeks to utilize platforms to reach especially younger audiences (Stollfuß, 2024). In contrast, the Norwegian example showed a strategical, albeit partial, withdrawal or consolidation of offers on social media platforms, aimed to help strengthen the public service media institution's own domain.

Based on a presentation of these two cases, and a closer look at informants’ reasoning about the attractiveness of the Norwegian NRK's news website, the article has sought to compare on a larger scale the context for different European public service media, concerning trust and power vis-à-vis platforms, gauged through survey data on preferences for social media as gateways to news. This has provided an opportunity to compare the basis, so to speak, for different organizations’ relational power, and their specific spaces of negotiation facing platforms. While the short discussion presented here provides only a start, insight into how routine use of social media platforms for news, and how users mainly get to news, brought out how regional differences in the standing of editorial news providers – public service ones included – significantly differ. Extending the argument by Poell et al. (2023), the article demonstrates how media systems and societal contexts matter for these spaces of negotiation, even when focusing on one particular type of media organization such as public service media.

## Notes

1. Survey samples are based on self-recruited panels, where respondents were invited to participate measured against quotas based on age, gender and region (Eastbury, 2022). An advantage of using panels is the high motivation levels among the respondents; a disadvantage is that the samples are prone to biases, since certain types of people are more likely to volunteer for inclusion and longevity in such panels. In the present survey, this challenge is mitigated through the sampling as well as through weighting (Eastbury, 2022). The samples reflect the part of the population in each country that has Internet access. This is found to disfavour less affluent and older segments of the population, as well as those with low levels of formal education.
